# Carelli on art ‘The Dali Universe’

**DOI:** 10.1080/17571472.2017.1364743

**Published:** 2017-08-13

**Authors:** Francesco Carelli

**Affiliations:** Family Medicine, University of Milan, Milan, Italy

**Keywords:** Multimedia, surreal

## Abstract

Interactive and multimedia methods are the future, already developing in teaching and learning modules in Medicine. It happens and has to happen because:

1) It improves tools for the teacher in preparing and showing the bulk of materials on which students must study and learn; it enables interactivity with learners both in formative pathway and in final assessment.

2) It improves attention, interest and involvement of students and learners, and acts as a guideline to progressive broadening in researches, studies, considerations, and information exchanges among different learners.

3) The rapid progress in application of technology, WEB area and sources researches allows such a deepening and widening interactivity that before was never possible to imagine. It is the same thing also in CME, sometimes so dry, and dropped from above, while the new methodology may/must get rid of negative and boring features, often not useful in producing particular improvements in knowledge and quality.

The Professional Doctor needs to feel a protagonist part in situation to be investigated, where interaction with all what media tools can offer may produce an “educative” improvement, much faster, effective and pleasant/rewarding.

## Why this matters to me

Art and medicine are linked in many ways, and this piece focusses on its possible role in medical education. My thoughts are as ever personal.

## Key message

Innovation in medical education methodology is always to be encouraged

This exhibition, at Palazzo Belloni in Bologna, Italy from 25 November 2016 to 7 May 2017, is a new, ‘diffuse’ and immersive cultural experience.

About 200 works from the collection *The Dalì Universe* (one of the richest documentations of Dalì’s artistic story) are the protagonists of an interactive and multimedia journey inviting visitors to an involving, participative experience.

There are 22 museum sculptures comprising 10 glass works made in the late 1960s [in collaboration with the famous *Daum* crystal factory in Nancy] and 12 gold objects; as well as more than 100 graphics from illustrated books and 4 monumental sculptures placed in strategical locations around the city.

The creative act of this unconventional curatorial work involves all aspects of the exhibition, as if they were a related whole. The aim is to be sympathetic to the artist in transmitting to the public an emotional message, rather than a didactic one. Multimedia and interactivity become an integral part of the tale.

Visitors can go deep into the labyrinth of the artist’s polymeric mind to discover the creative results of his imagination. It is a journey through multi-dimensions, from the bi-dimensionality of graphics, through tri-dimensionality of sculptures, to the virtual fourth dimension of space and time.

Spectators are prompted again and again to use all their senses to interact with *Dalì Universe*, where his works are set up so to communicate with interactive installations (3D animation, immersive projections) in a tour of continued discovery and surprise.

In the exhibition pathway, we discover the soul of the artist, whose creative contributions not only join with surrealist painting but touch the most different and fertile ambits of twentieth century culture: from cinema to fashion, from design to advertising, from literature to cooking, and from psychoanalysis to the physics of particles.

The idea, and the challenge, is to imagine the exhibition spaces as containers, fluid inside and opened towards outside, in harmony with the territory. They could be described as propulsive centres from which culture spreads by centrifugal force involving the town to make it participant and protagonist.

For this reason, surreal appointments and events at 360 degrees are programmed or occur by surprise in unpredictable places, in partnership with local institutions or organizations, in continuous relationship outside/inside, online/offline and real/virtual.

For instance, an app of augmented reality [#Dali experience] is at visitors’ disposal to see a townscape with Dali’s ‘look’, and in the process discovering his strange objects spread all over this territory, perhaps taking pictures to share on a social network.

These kinds of interactive and multimedia methods are arguably the future; already developing in teaching and learning modules in medicine. It has to happen because:

• It can improves tools teachers in the preparing and delivering the bulk of material through which students study and learn.
• It enables interactivity with learners both in formative learning and in assessment.
• It can improve attention, interest and involvement of students and learners, and acts as a guideline to progressive broadening in research, study, and information exchanges among different learners.
• The rapid progress in application of technology, web features and sources researches allows such a deepening and widening interactivity that before was never possible to imagine.
• Continuing Medical Education can be accused of being sometimes too dry, or didactic. Using new methodology can arguably be used to reduce potentially negative or boring features.

Thus the Doctor as educator should feel as a protagonist in this new educative environment where interaction with all these media tools can offer should be the norm. Dali shows us the way.

## Disclosure statement

No potential conflict of interest was reported by the author.

